# Survival Trends and Prognostic Modeling in ALK‐Positive Anaplastic Large Cell Lymphoma: A Population‐Based Study in the Brentuximab Vedotin Era

**DOI:** 10.1002/cam4.71695

**Published:** 2026-03-06

**Authors:** Qiuyu Zhang, Yanyan Liu

**Affiliations:** ^1^ Department of Internal Medicine The Affiliated Cancer Hospital of Zhengzhou University & Henan Cancer Hospital Zhengzhou China

**Keywords:** ALK‐positive anaplastic large cell lymphoma, brentuximab vedotin, machine learning, random survival forest, SEER database

## Abstract

**Background:**

ALK‐positive anaplastic large cell lymphoma (ALK+ ALCL) is a rare subtype of peripheral T‐cell lymphoma with traditionally favorable prognosis. The introduction of brentuximab vedotin (BV) has significantly impacted treatment outcomes, but the long‐term survival trends and predictive factors for this population remain underexplored.

**Methods:**

A total of 1548 patients diagnosed with ALK+ ALCL between 2004 and 2017 were identified from the Surveillance, Epidemiology, and End Results (SEER) database. Patients were categorized into two eras: pre–BV (2004–2010, *n* = 795) and post–BV (2011–2017, *n* = 753). Overall survival (OS) was compared between eras. A random survival forest (RSF) model was constructed to identify prognostic factors and stratify survival risk in the post–BV cohort.

**Results:**

OS significantly improved in the post–BV era (HR = 0.68, 95% CI: 0.58–0.81, *p* < 0.001), with the 5‐year OS increasing from 59.3% to 72.3%. The RSF model identified age, Ann Arbor stage, primary site, B symptoms, and radiotherapy as key prognostic factors, showing good discrimination with C‐indices of 0.775 (training cohort) and 0.728 (testing cohort). Notably, radiotherapy was found to be a protective factor. The model effectively stratified patients into high‐risk (5‐year OS: 49.3%) and low‐risk (86.0%) groups.

**Conclusion:**

The introduction of BV has significantly improved real‐world survival in ALK+ ALCL. The RSF model enables individualized risk stratification and may support future precision treatment strategies.

## 1 Introduction

ALK‐positive anaplastic large cell lymphoma (ALK+ ALCL) represents about 3% of all non‐Hodgkin lymphomas [1, 2]. It is considered a rare subtype within peripheral T‐cell lymphoma (PTCL). It predominantly impacts adults in their younger to middle years, with a greater prevalence seen in younger individuals [3, 4]. Its hallmark is the NPM1–ALK fusion [5, 6], conferring distinct molecular characteristics [7]. Compared to other PTCL subtypes, ALK+ ALCL generally exhibits superior clinical outcomes following standard chemotherapy [8, 9]. However, a subset of patients continues to experience suboptimal outcomes under conventional treatment [8, 10].

Recently, brentuximab vedotin (BV) has shown notable effectiveness in treating relapsed or refractory systemic ALCL. BV consists of an anti‐CD30 monoclonal antibody conjugated to a cytotoxic agent. This design enables selective targeting of CD30‐positive tumor cells, leading to apoptosis [11, 12, 13]. It may also enhance antitumor immunity, contributing to its therapeutic effect [14, 15]. In 2011, the U.S. Food and Drug Administration (FDA) authorized BV for treating relapsed or refractory systemic ALCL [16], following key findings from a phase II trial [17]. Since its regulatory approval, BV has been increasingly adopted in clinical practice and is now incorporated into frontline therapeutic regimens with growing frequency [18, 19]. Despite these advances, comprehensive real‐world evidence regarding long‐term survival outcomes and prognostic determinants in the era following BV approval remains limited, particularly for the rare ALK+ ALCL subtype. Robust population‐based studies are therefore critical to delineate contemporary survival trends and to identify high‐risk subgroups with limited response to BV‐based therapies, thereby informing risk‐adapted treatment strategies.

To address this gap, we used the Surveillance, Epidemiology, and End Results (SEER) database to construct a cohort of ALK+ ALCL patients. We compared survival outcomes between the pre– and post–BV eras and developed a predictive model using the random survival forest (RSF) algorithm for post‐BV patients. Our aim was to identify prognostic factors and stratify survival risk to aid in clinical decision‐making and tailored treatment strategies.

## 2 Materials and Methods

### 2.1 Data Sources and Cohort

This research used data from the SEER database. The SEER program collects information on cancer incidence, survival rates, and related clinical factors, representing about 45.9% of the U.S. population [20]. Patient data were retrieved from the SEER database (Incidence‐SEER Research Data, 17 Registries, Nov 2023 Sub (2000–2021)) using SEER*Stat software (version 8.4.5). The inclusion criteria were [1] diagnosis of ALK+ ALCL based on the ICD‐O‐3 (histology code: 9714/3) [2] diagnosis between 2004 and 2017 [3] age at diagnosis between 20 and 79 years [4] ALK+ ALCL as the patient's first primary malignancy. Exclusion criteria were: unknown survival time.

### 2.2 Variable Definition and Management

The variables collected for this study encompassed demographic factors (such as age, sex, and race), disease characteristics (including primary site, Ann Arbor stage, and B symptoms), and treatment details (chemotherapy and radiotherapy). Based on the year BV was approved in the United States (2011) [16], patients were classified into two temporal cohorts: the pre–BV era (2004–2010, *n* = 795) and the post–BV era (2011–2017, *n* = 753). The 20–79‐year range focused on adult ALK+ ALCL, excluding pediatric cases (< 20 years) due to distinct biology and treatments, and patients ≥ 80 years due to comorbidities and less intensive therapies, ensuring robust sample size and data homogeneity [21]. To convert age into a categorical variable, we referred to the epidemiological characteristics and prognostic patterns of ALK+ ALCL. Cutoff points at 40 and 60 years were selected to group patients into 20–39, 40–59, and 60–79 years. The 40‐year threshold reflects the disease's predilection for younger individuals and its typically favorable prognosis in this population [22], while the 60‐year threshold aligns with the high‐risk age cutoff commonly used in the International Prognostic Index (IPI) [23]. Primary site was categorized into three groups according to SEER topography codes: lymph node, skin, and other extranodal sites (excluding skin). The “skin” group represents cases coded as having skin involvement as the primary site, but does not imply a diagnosis of primary cutaneous ALCL, which is classified under a different histology code and was excluded from this study. Missing data for race (1.3%), primary site (0.4%), Ann Arbor stage (16.7%), and B symptoms (16.3%) were imputed using a random forest algorithm, which predicts missing values based on patterns in other variables to minimize bias.

### 2.3 Machine Learning Model Construction

To identify key prognostic factors and enable risk stratification for patients diagnosed with ALK+ ALCL in the post–BV era, we developed a machine learning–based predictive model using the RSF algorithm. RSF is a nonparametric approach that handles right‐censored survival data and models complex nonlinear associations between covariates and survival outcomes. Given the rarity and heterogeneity of ALK+ ALCL, RSF offers advantages over traditional Cox regression models in terms of robustness and flexibility. In the post–BV cohort (2011–2017), patients were divided into a training set (70%, *n* = 528) for model development and a testing set (30%, *n* = 225) for validation. To assess the model's ability to discriminate, Harrell's concordance index (C‐index) was employed. Using predicted risk scores, patients were classified into high‐ and low‐risk groups. Kaplan–Meier analysis was then used to assess survival differences. Variable importance (VIMP) scores were also computed to quantify the impact of individual predictors and highlight key factors influencing prognosis after BV approval.

### 2.4 Statistical Analysis

To compare baseline characteristics between groups, the Chi‐square test or Fisher's exact test was used, depending on the nature of the data. Overall survival (OS) was defined as the time from diagnosis to death or last follow‐up. Kaplan–Meier curves were used to estimate survival probabilities. The log‐rank test was then applied to assess differences. The median follow‐up time was determined through the Reverse Kaplan–Meier method, which accounts for censored data. Subgroup analyses by diagnostic era were conducted to evaluate survival differences across clinical variables. Interaction terms were then added to Cox models to assess whether the effect of the BV era varied among subgroups. Univariate and multivariate Cox regression analyses were conducted to identify key factors related to OS. Model discrimination was assessed using the C‐index and the area under the receiver operating characteristic curve (AUC). Calibration was assessed using calibration plots. Clinical usefulness was then evaluated through decision curve analysis (DCA). Sensitivity analyses were performed by truncating follow‐up at 60, 84, and 86 months in Kaplan–Meier and Cox models to assess the impact of differential follow‐up duration. Imputation robustness was confirmed through distribution comparisons between observed and imputed values, along with sensitivity analyses contrasting imputation results and complete cases. All statistical tests were two‐sided, and *p*‐values less than 0.05 were considered significant. R software (version 4.4.2) was used for all statistical analyses.

## 3 Results

### 3.1 Study Cohort Baseline Characteristics

**FIGURE 1:**
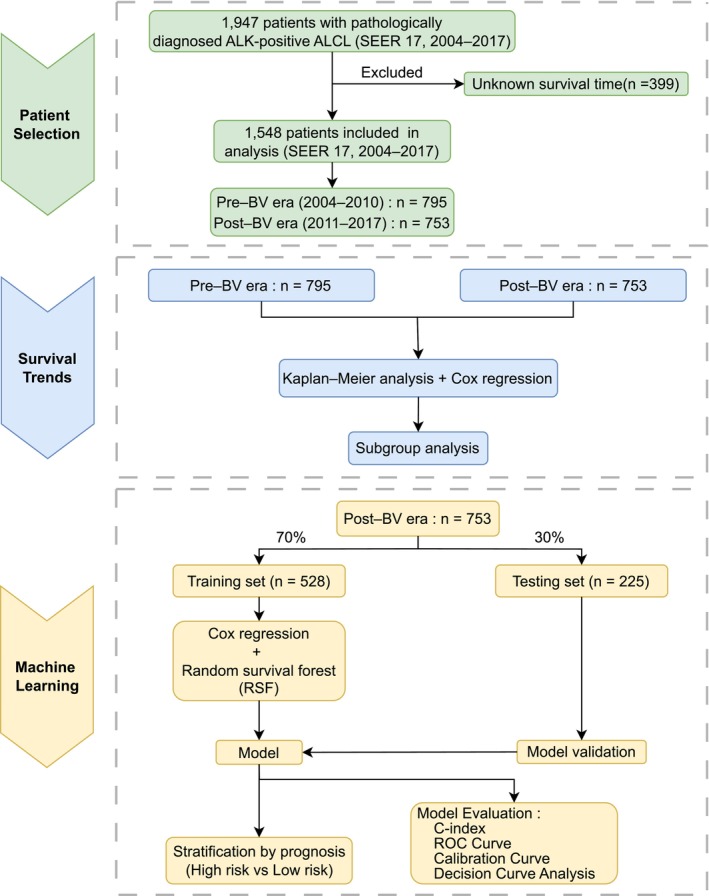
Flowchart of patient selection and study design. Abbreviations: ALCL, anaplastic large cell lymphoma; SEER, Surveillance, Epidemiology, and End Results; brentuximab vedotin, BV; C‐index, concordance index; ROC curve, receiver operating characteristic curve.

A total of 1548 patients diagnosed with ALK+ ALCL were included according to predefined criteria (Figure 1 and Table S1). Patients were categorized into the pre–BV era (2004–2010, *n* = 795) and post–BV era (2011–2017, *n* = 753). Baseline characteristics are summarized in Table 1. The median follow‐up for the entire cohort was 123 months. It was 171 months in the pre–BV era and 86 months in the post–BV era. Significant differences were observed in racial distribution (*p* = 0.005) and use of radiotherapy (*p* = 0.022). Specifically, White patients represented 83% of the pre–BV cohort and 76% of the post–BV cohort, while the proportion of Black patients increased from 11% to 16%. Radiotherapy was used more frequently in the pre–BV era (23% vs. 18%). Other variables showed no significant differences (all *p* > 0.05).

**TABLE 1:** Demographic and clinical features of patients in the pre–BV and post–BV eras.

Characteristic	Pre–BV era (*n* = 795)	Post–BV era (*n* = 753)	*P*
Age, *n* (%)			0.218
20–39 years	214 (27)	223 (30)	
40–59 years	325 (41)	276 (37)	
60–79 years	256 (32)	254 (34)	
Sex, *n* (%)			0.067
Female	284 (36)	304 (40)	
Male	511 (64)	449 (60)	
Race, *n* (%)			0.005
White	658 (83)	574 (76)	
Black	87 (11)	119 (16)	
Others	50 (6)	60 (8)	
Primary site, *n* (%)			0.178
Lymph node	591 (74)	586 (78)	
Skin	73 (9)	52 (7)	
Others	131 (16)	115 (15)	
Ann Arbor stage, *n* (%)			0.195
I	209 (26)	192 (25)	
II	192 (24)	153 (20)	
III	156 (20)	152 (20)	
IV	238 (30)	256 (34)	
Radiotherapy, *n* (%)			0.022
No/Unknown	613 (77)	617 (82)	
Yes	182 (23)	136 (18)	
Chemotherapy, *n* (%)			0.52
No/Unknown	171 (22)	151 (20)	
Yes	624 (78)	602 (80)	
B symptoms, *n* (%)			0.134
No	426 (54)	433 (58)	
Yes	369 (46)	320 (42)	

Abbreviation: BV, brentuximab vedotin.

### 3.2 Temporal Survival Trends

**FIGURE 2:**
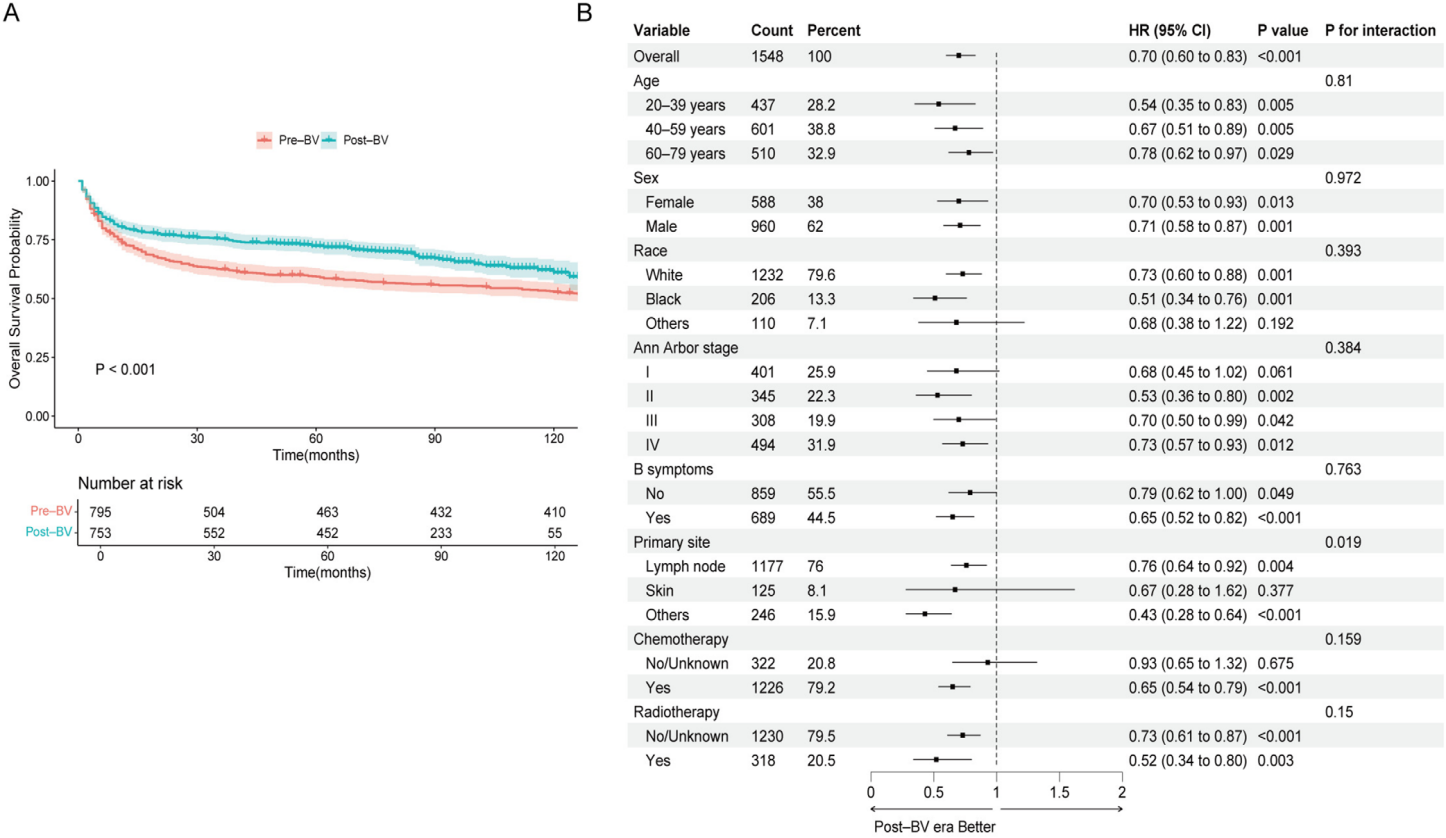
Overall survival and subgroup analysis across brentuximab vedotin (BV) eras. (A) Kaplan–Meier curves comparing overall survival (OS) between the pre–BV (2004–2010) and post–BV (2011–2017) eras in the entire cohort (HR = 0.70, 95% CI: 0.60–0.83; *p* < 0.001). (B) Forest plot of subgroup analyses comparing OS between the two eras across baseline characteristics.

OS significantly improved in the post–BV era compared to the pre–BV era (HR = 0.70, 95% CI: 0.60–0.83, *p* < 0.001; Figure 2A). The median survival time in the pre–BV era was 147 months, whereas it has not been reached in the post–BV era. The 5‐year OS rate increased from 59.3% to 72.3%, suggesting a meaningful survival benefit associated with BV introduction. Sensitivity analyses truncating follow‐up at 60, 84, and 86 months confirmed the post‐BV survival benefit remained significant (log‐rank *p* < 0.001; Table S2).

Although Kaplan–Meier analysis indicated a survival improvement in the post–BV era, it did not consider other clinical factors that could impact outcomes. Therefore, a multivariable Cox regression was used to adjust for confounders, including age, sex, race, primary site, Ann Arbor stage, B symptoms, chemotherapy, and radiotherapy (Table S3). After adjusting for potential confounders, the results showed that diagnosis in the post–BV era was independently linked to improved OS (HR = 0.68, 95% CI: 0.58–0.81, *p* < 0.001). These findings suggest that survival improvement is likely attributed to the introduction of BV and advancements in treatment strategies.

### 3.3 Subgroup Analyses

**FIGURE 3:**
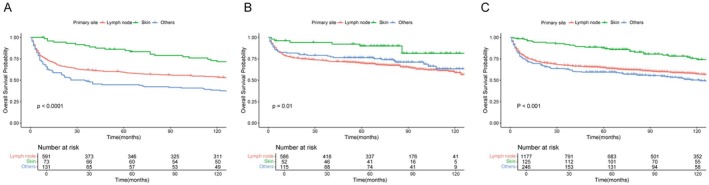
Kaplan–Meier survival curves for different primary sites in the (A) pre–BV era (2004–2010), (B) post–BV era (2011–2017), and (C) overall cohort (2004–2017). Abbreviations: BV, brentuximab vedotin.

Subgroup analyses confirmed that the survival benefit in the post–BV era was broadly consistent across most clinical subgroups, with no significant interactions observed (Figure 2B). However, the survival benefit varied by primary site. Patients with lymph node (HR = 0.76, 95% CI: 0.64–0.92, *p* = 0.004) or other extranodal involvement (HR = 0.43, 95% CI: 0.28–0.64, *p* < 0.001) showed significant improvement, whereas those with skin involvement did not (HR = 0.67, 95% CI: 0.28–1.62, *p* = 0.377; interaction *p* = 0.019). OS curves showed that skin‐involved patients consistently exhibited better survival than those with other primary sites, regardless of the era (Figure 3A–C). Among skin‐involved patients, the 5‐year OS rates were 86.1% in the pre–BV era and 90.0% in the post–BV era (*p* = 0.51). These findings suggest that the limited survival benefit from BV in this subgroup is likely due to their already favorable baseline prognosis.

### 3.4 Baseline Characteristics of the Training and Testing Cohorts

The post–BV cohort (*n* = 753) was randomly split into a training set (*n* = 528) and a testing set (*n* = 225) at a 7:3 ratio (Figure 1). The two groups showed similar baseline characteristics (all *p* > 0.05; Table 2).

**TABLE 2:** Baseline characteristics of patients in the training and testing sets.

Characteristic	Training set (*n* = 528)	Testing set (*n* = 225)	*P*
Age, *n* (%)			0.945
20–39 years	155 (29)	68 (30)	
40–59 years	193 (37)	83 (37)	
60–79 years	180 (34)	74 (33)	
Sex, *n* (%)			0.828
Female	215 (41)	89 (40)	
Male	313 (59)	136 (60)	
Race, *n* (%)			0.835
White	400 (76)	174 (77)	
Black	84 (16)	35 (16)	
Others	44 (8)	16 (7)	
Primary site, *n* (%)			0.14
Lymph node	402 (76)	184 (82)	
Skin	42 (8)	10 (4)	
Others	84 (16)	31 (14)	
Ann Arbor stage, *n* (%)			0.867
I	131 (25)	61 (27)	
II	109 (21)	44 (20)	
III	105 (20)	47 (21)	
IV	183 (35)	73 (32)	
Radiotherapy, *n* (%)			0.814
No/Unknown	431 (82)	186 (83)	
Yes	97 (18)	39 (17)	
Chemotherapy, *n* (%)			0.747
No/Unknown	108 (20)	43 (19)	
Yes	420 (80)	182 (81)	
B symptoms, *n* (%)			0.985
No	303 (57)	130 (58)	
Yes	225 (43)	95 (42)	

### 3.5 Feature Selection and Model Construction

**FIGURE 4:**
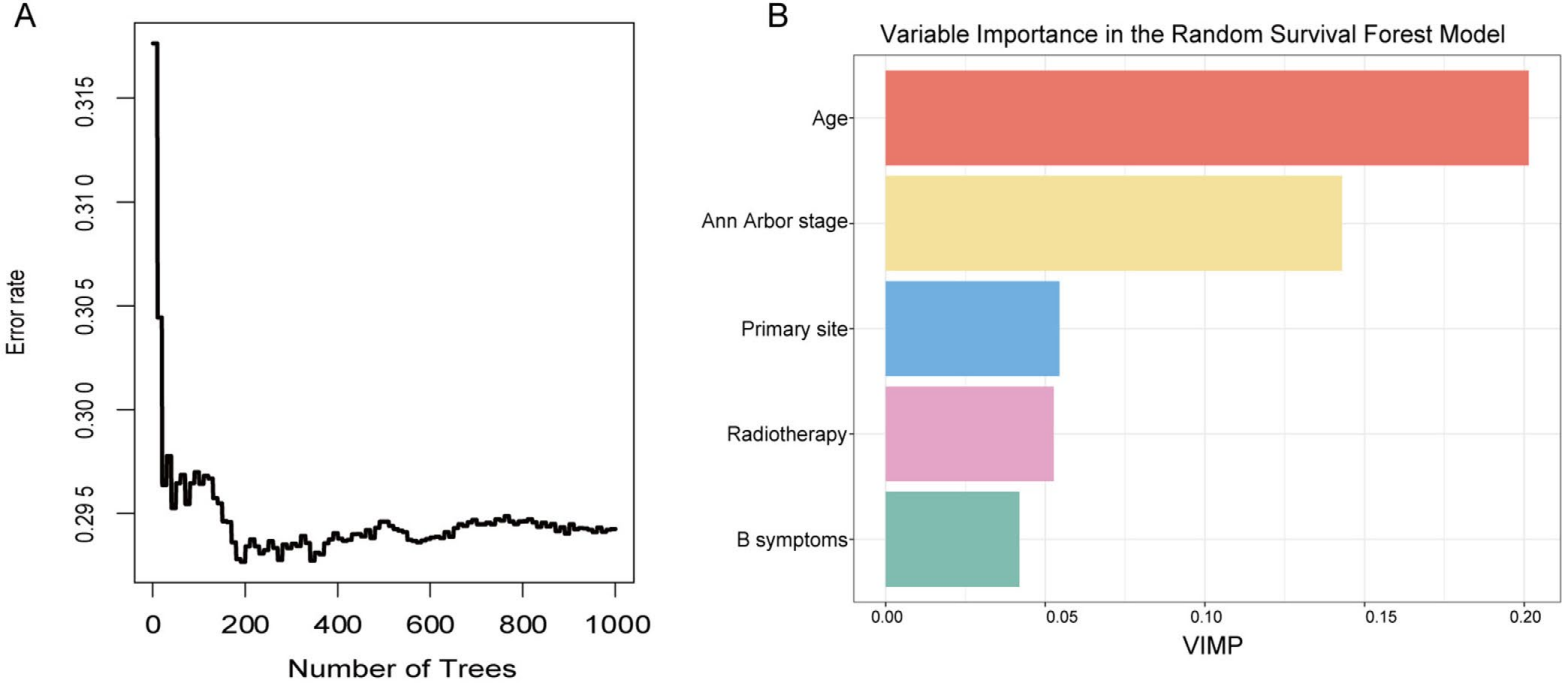
Random survival forest model performance and variable importance. (A) Prediction error rate curve across increasing number of trees (*N* = 1000). (B) Variable importance ranking based on out‐of‐bag data.

Univariate Cox regression identified five variables significantly associated with OS: age, Ann Arbor stage, primary site, radiotherapy, and B symptoms (Table 3). These factors trained the model on the training set and were then used to validate it on the testing set. Multivariate results confirmed age, stage, and B symptoms as adverse prognostic factors, with radiotherapy being protective. Five variables with *p* < 0.05 in univariable Cox regression—including age, primary site, Ann Arbor stage, radiotherapy, and B symptoms—were included in the RSF model. A total of 1000 survival trees were grown (ntree = 1000), with the minimum terminal node size set to 8 (nodesize = 8). The out‐of‐bag (OOB) error rate decreased and stabilized after approximately 200 trees were grown (Figure 4A). At each node split, two variables were randomly chosen (mtry = 2), with the log‐rank test applied as the criterion for splitting (splitrule = ‘logrank’). Variable importance and proximity were calculated during training. A random seed of 123 was specified to ensure reproducibility. Model performance was evaluated using OOB estimates, yielding a standardized continuous ranked probability score (CRPS) of 0.166 and an OOB prediction error of 0.294.

**TABLE 3:** Univariate and multivariate Cox regression results for patients in the post–BV era training set.

Characteristic	Univariate analysis		Multivariate analysis	
	HR (95% CI)	*p*	HR (95% CI)	*p*
Age				
20–39 years				
40–59 years	2.41 (1.44–4.01)	< 0.001	2.71 (1.62–4.52)	< 0.001
60–79 years	4.75 (2.92–7.72)	< 0.001	5.36 (3.28–8.75)	< 0.001
Sex				
Female				
Male	1.24 (0.90–1.70)	0.183		
Race				
White				
Black	1.11 (0.74–1.67)	0.603		
Others	1.09 (0.63–1.90)	0.761		
Primary site				
Lymph node				
Skin	0.30 (0.12–0.72)	0.007	0.44 (0.17–1.11)	0.082
Others	0.78 (0.50–1.21)	0.264	1.05 (0.66–1.66)	0.837
Ann Arbor stage				
I				
II	1.18 (0.66–2.10)	0.583	1.20 (0.66–2.18)	0.545
III	2.37 (1.42–3.95)	< 0.001	1.85 (1.07–3.19)	0.027
IV	3.14 (1.99–4.96)	< 0.001	2.35 (1.44–3.84)	< 0.001
Radiotherapy				
No/Unknown				
Yes	0.41 (0.25–0.69)	< 0.001	0.56 (0.33–0.95)	0.031
Chemotherapy				
No/Unknown				
Yes	0.94 (0.65–1.36)	0.731		
B symptoms				
No				
Yes	1.65 (1.21–2.24)	0.001	1.40 (1.01–1.93)	0.041

Abbreviation: BV, brentuximab vedotin.

VIMP scores were derived from OOB data to assess each predictor's relative contribution to model performance. As shown in Figure 4B, age was the most important predictor (VIMP = 0.2006), followed by Ann Arbor stage (0.1421). The VIMP scores of primary site (0.0548), radiotherapy (0.0525), and B symptoms (0.0416) were relatively lower.

### 3.6 Risk Stratification Using the Model

**FIGURE 5:**
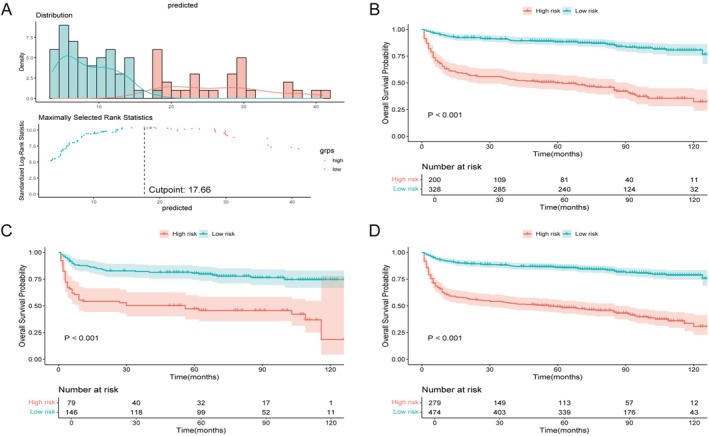
Risk stratification using the random survival forest (RSF) model. (A) Determination of the optimal cut‐off risk score (17.66) derived from the RSF model (B–C) Overall survival (OS) curves stratified by the cut‐off in the training (B) and testing (C) cohorts of the post–BV era (2011–2017). (D) OS curves for all post–BV patients stratified by the same cut‐off score. Abbreviations: BV, brentuximab vedotin.

Patients were classified into high‐ and low‐risk groups based on the optimal cut‐off score of 17.66 from the RSF model (Figure 5A). This classification was applied to both the training and testing cohorts (Figure 5B,C). Kaplan–Meier analysis indicated that OS was significantly reduced in the high‐risk group when compared to the low‐risk group (*p* < 0.001). The same cut‐off was then applied to all post–BV patients for final stratification (Figure 5D). Table 4 provides a summary of the clinical characteristics for both risk groups. In the high‐risk group (*n* = 279), the median survival time was 56 months, while it was not reached in the low‐risk group (*n* = 474), with 5‐year OS rates of 49.3% and 86.0%, respectively. Key adverse prognostic factors in the high‐risk group included older age (≥ 60 years), advanced Ann Arbor stage (III or IV), non‐cutaneous primary site, and B symptoms, while radiotherapy was a protective factor. Kaplan–Meier curves stratified by these variables further confirmed their prognostic relevance (Figure S1A–E).

**TABLE 4:** Clinical characteristics of high‐risk and low‐risk patients in the post–BV era as defined by the prognostic model.

Characteristic	Low risk (*n* = 474)	High risk (*n* = 279)	*P*
Age, *n* (%)			< 0.001
20–39 years	217 (46)	6 (2)	
40–59 years	198 (42)	78 (28)	
60–79 years	59 (12)	195 (70)	
Sex, *n* (%)			0.087
Female	203 (43)	101 (36)	
Male	271 (57)	178 (64)	
Race, *n* (%)			0.388
White	367 (77)	207 (74)	
Black	74 (16)	45 (16)	
Others	33 (7)	27 (10)	
Primary site, *n* (%)			< 0.001
Lymph node	350 (74)	236 (85)	
Skin	46 (10)	6 (2)	
Others	78 (16)	37 (13)	
Ann Arbor stage, *n* (%)			< 0.001
I	168 (35)	24 (9)	
II	123 (26)	30 (11)	
III	78 (16)	74 (27)	
IV	105 (22)	151 (54)	
Radiotherapy, *n* (%)			< 0.001
No/Unknown	354 (75)	263 (94)	
Yes	120 (25)	16 (6)	
Chemotherapy, *n* (%)			0.16
No/Unknown	103 (22)	48 (17)	
Yes	371 (78)	231 (83)	
B symptoms, *n* (%)			< 0.001
No	304 (64)	129 (46)	
Yes	170 (36)	150 (54)	

Abbreviation: BV, brentuximab vedotin.

### 3.7 Model Performance Evaluation

**FIGURE 6:**
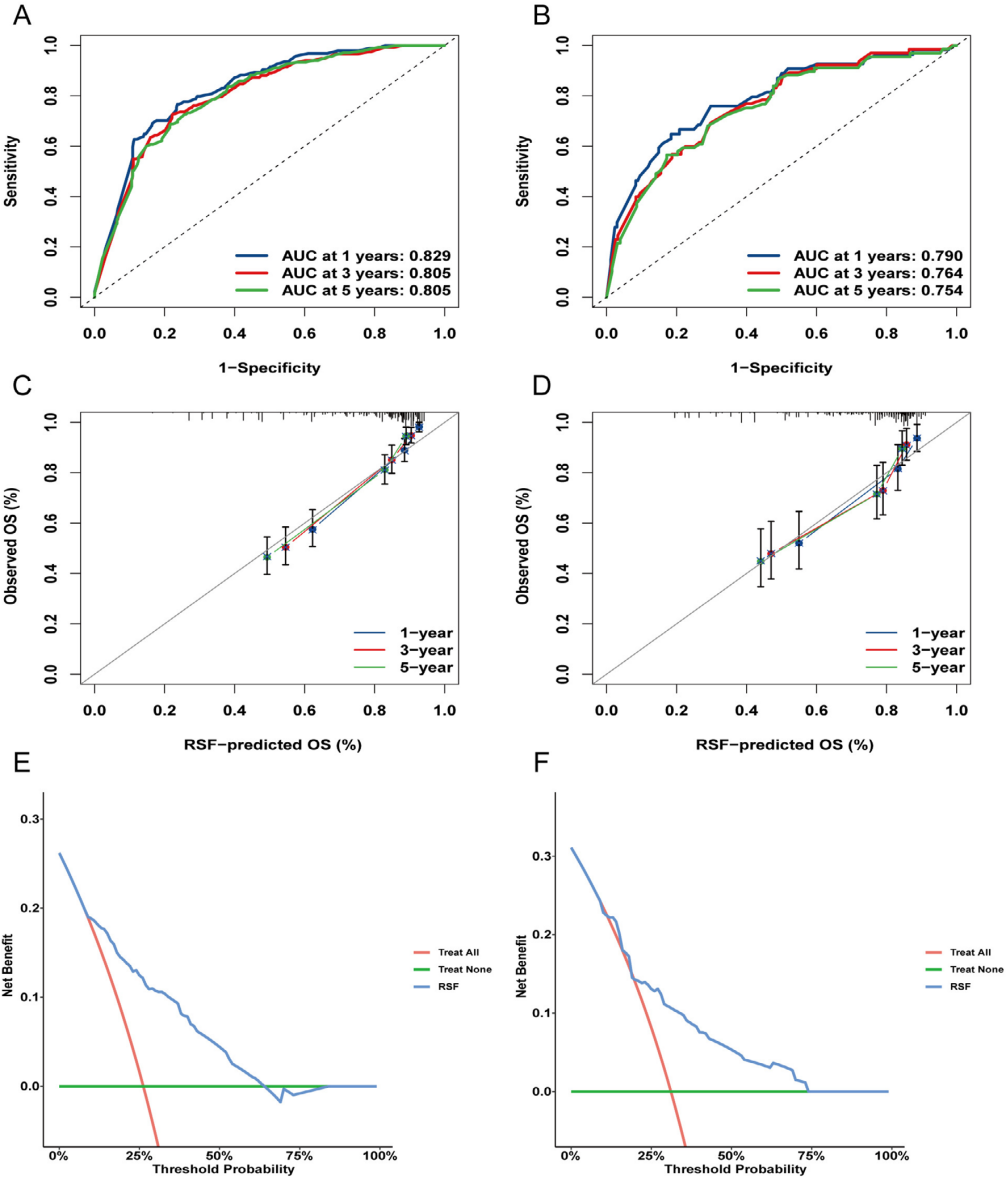
Discrimination, calibration, and clinical utility of the model. (A–B) Receiver operating characteristic (ROC) curves for 1‐, 3‐, and 5‐year overall survival (OS) in the training and testing cohorts (C–D). Calibration plots for 1‐, 3‐, and 5‐year OS in the training and testing cohorts (E–F). Decision curve analysis (DCA) for 5‐year OS in the training and testing cohorts.

The RSF model demonstrated strong discrimination, with C‐indexes of 0.775 in training and 0.728 in testing. For 1‐, 3‐, and 5‐year OS, the AUCs were 0.829, 0.805, and 0.805 in the training cohort, and 0.790, 0.764, and 0.754 in the testing cohort (Figure 6A,B). These results demonstrate stable predictive performance across all timepoints. Calibration plots showed that predicted and observed survival probabilities were closely aligned for all timepoints in both cohorts (Figure 6C,D), suggesting good model calibration. The DCA curves for 1‐, 3‐, and 5‐year OS (Figures S2A–D and 6E,F) revealed a higher net benefit. This comparison with the ‘treat‐all’ and ‘treat‐none’ strategies highlights the model's clinical utility. Overall, the RSF model showed strong predictive accuracy, reliable calibration, and potential clinical applicability.

## 4 Discussion

In this retrospective study using the SEER database, we assessed survival trends in ALK+ ALCL patients before and after the approval of BV. OS improved significantly in the post–BV era, even after adjustment for clinical covariates, suggesting that the widespread adoption of BV likely contributed to this improvement. A machine learning–based RSF model developed in the post–BV cohort enabled effective survival risk stratification and showed strong predictive performance.

Our results show a significant survival improvement for ALK+ ALCL patients since the introduction of BV, even after adjusting for confounders like age, disease stage, and treatment modality. The expanding use of BV following its FDA approval in 2011 likely underlies this improvement [16, 18], consistent with prior reports of its efficacy and safety [17, 19]. The landmark phase III ECHELON‐2 trial reported a 5‐year OS of approximately 75.8% in the BV‐containing arm versus 68.7% in the traditional chemotherapy arm among patients with systemic ALCL [24]. In our population‐based cohort of ALK+ ALCL, the 5‐year OS improved from 59.3% in the pre‐BV era to 72.3% in the post‐BV era. Although direct comparison is limited by differences in patient selection and treatment settings, the population‐level survival gain observed in our study is consistent with the efficacy demonstrated in ECHELON‐2, reinforcing the broader clinical impact of BV availability. Our study utilizes the SEER database, which includes data representing approximately 45.9% of the U.S. population [20]. This allows us to provide robust, population‐level evidence with strong external validity. Additionally, our study included 1548 ALK‐positive ALCL patients diagnosed between 2004 and 2017, representing a substantial sample size for this rare disease and a follow‐up period of up to 123 months, enabling robust assessment of long‐term survival trends. To date, real‐world studies examining the impact of BV in ALK+ ALCL remain limited and often involve small sample sizes [25, 26, 27]. By leveraging a large cohort with extended follow‐up, we address a major gap in the current literature.

Subgroup analyses confirmed that BV improved survival across most patient groups. However, the benefit was limited in those with skin involvement, likely due to their already favorable baseline prognosis (Figure 3A–C). Although this subgroup demonstrated prolonged survival regardless of treatment era, the finding remains clinically meaningful. Literature on systemic ALK+ ALCL with cutaneous involvement is limited to small series or case reports, lacking population‐level validation [28, 29]. By including a substantial number of such cases, our study provides real‐world evidence on this understudied group and supports the development of more individualized treatment approaches.

To identify high‐risk subgroups in the post–BV era, we developed a RSF model using real‐world data. The model identified five key prognostic factors—age, Ann Arbor stage, primary site, radiotherapy, and B symptoms—and demonstrated strong survival discrimination. This is the first large‐scale, machine learning‐based prognostic model developed specifically for ALK+ ALCL, a rare subtype. Unlike previous studies, which have not explored such modeling for this population, our approach is novel, outperforms traditional IPI (C‐index 0.728 vs. 0.60–0.70) [30, 31], and offers clinical potential. The RSF model integrates multiple risk factors for individualized survival prediction, guiding treatment decisions and follow‐up strategies. With further validation, it could be incorporated into clinical decision support systems, providing real‐time, data‐driven risk stratification in routine clinical practice.

The results indicated that age and Ann Arbor stage were the most significant predictors of OS, aligning with findings from large‐scale studies in ALCL and other PTCLs [32]. These two factors are key components of the IPI, a widely used prognostic tool in lymphoma. This suggests that, despite substantial therapeutic progress, host‐related factors and baseline disease burden remain crucial in shaping long‐term outcomes.

Primary site was also a major prognostic variable, underscoring the heterogeneity across anatomical presentations. Patients with skin involvement demonstrated favorable baseline prognosis (Figure 3A–C), showing limited additional benefit from BV. In contrast, patients with lymph node or other extranodal involvement showed significant survival improvements with BV. The 5‐year OS increased from 59.3% to 70% in lymph node patients and from 44.8% to 76.3% in extranodal patients. This highlights the importance of personalized treatment strategies based on primary site.

B symptoms, such as fever, night sweats, and weight loss, are often linked to systemic inflammation and immune response [33]. Traditionally, B symptoms have been considered important prognostic factors in lymphoma. They are typically caused by inflammatory cytokines or metabolic disturbances released by tumor cells, reflecting the biological activity of the lymphoma [34]. In our model, the VIMP of B symptoms was relatively low, suggesting that the introduction of immune therapies like BV may have mitigated their prognostic impact. Therefore, for patients with B symptoms, more aggressive treatment options, such as regimens combining BV with other agents like ALK inhibitors, should be considered.

In contrast, radiotherapy ranked higher in VIMP within the RSF model and was identified as a protective factor. Traditionally considered an adjunct for local control, radiotherapy is often omitted in patients with good chemotherapy response to reduce long‐term toxicity [35, 36]. However, recent population‐based evidence in limited‐stage PTCL, including ALK+ ALCL, suggests otherwise: adding radiotherapy to chemotherapy significantly improves 5‐year OS compared to chemotherapy alone (72% vs. 55%) [37]. Our findings align with this study, indicating that radiotherapy confers an independent survival advantage even in the context of BV‐based treatment (Figure 4B and Figure S1D). This observation supports further prospective studies to evaluate the potential synergy between radiotherapy and BV.

The identified variables play a central role in predicting outcomes and enabling risk stratification. Classifying patients as high‐risk or low‐risk may assist in tailoring personalized treatment strategies. High‐risk patients, defined by factors such as age ≥ 60 years, advanced stage (III or IV), non‐cutaneous involvement, B symptoms, and absence of radiotherapy, showed significantly poorer survival and could benefit from more aggressive therapeutic approaches. For high‐risk patients, alternative or intensified treatment strategies may be warranted. Emerging targeted therapies, particularly ALK inhibitors, have shown promising efficacy in relapsed or refractory ALK+ ALCL [38, 39, 40]. Future studies should investigate the role of ALK inhibitors in frontline treatment to enhance outcomes and quality of life for this high‐risk subgroup.

## 5 Limitations

There are several limitations in this study. First, the SEER database is an epidemiological registry that does not contain detailed information on specific therapeutic agents. As such, the use of BV could not be directly identified but was instead inferred based on the calendar year of diagnosis. Nevertheless, the large sample size and population‐based nature of SEER provide a reasonable basis for this assumption. Second, some missing data may introduce residual uncertainty. To mitigate this, we applied random forest‐based imputation, which is robust for large datasets [41, 42], and further confirmed its validity through distribution diagnostics (Figure S3) and sensitivity analyses (Table S4). Third, the SEER database does not provide detailed cause of death information (e.g., disease relapse, progression, or treatment‐related toxicity). OS was therefore used as the study outcome, which remains the gold standard in population‐based oncology research. Finally, since ALK+ ALCL is a rare lymphoma, external validation of our findings in an independent cohort was not feasible. Prospective studies with detailed treatment data are needed to confirm these findings. Additionally, further refinement of risk stratification models should be pursued.

## 6 Conclusion

In conclusion, our large, population‐based study demonstrates that OS in ALK+ ALCL has significantly improved with the introduction of BV. We identified treatment benefit heterogeneity across subgroups and developed the first machine learning‐based prognostic model for this rare lymphoma subtype. The RSF model showed strong predictive performance. These findings fill gaps in real‐world evidence and offer a practical approach to risk stratification and personalized therapy. Prospective validation is needed to further optimize clinical decision‐making and improve outcomes in high‐risk patients.

## Author Contributions

Q.Z. conducted the study, analyzed the data, and drafted the manuscript. Y.L. supervised the study and revised the manuscript. Both authors approved the final version.

## Funding

This study was supported by the National Natural Science Foundation of China (82370198). The authors acknowledge the financial support provided by the Foundation.

## Ethics Statement

This study utilized publicly available, de‐identified data from the Surveillance, Epidemiology, and End Results (SEER) database. As such, institutional review board (IRB) approval and informed consent were not required, in accordance with the SEER Research Data Use Agreement.

## Conflicts of Interest

The authors declare no conflicts of interest.

## Supporting information


**Figure S1:** Kaplan–Meier curves for key prognostic factors identified by the model (A) Age group: 20–39, 40–59, and 60–79 years. (B) Ann Arbor stage: I, II, III, and IV. (C) Primary site: lymph node, skin, and others. (D) Radiotherapy: no/unknown vs. yes. (E) B symptoms: no vs. yes.

**Figure S2:** Additional decision curve analysis (DCA) of the model (A–B) DCA curves for 1‐year OS in the training and testing cohorts (C–D). DCA curves for 3‐year OS in the training and testing cohorts.

**Figure S3:** Diagnostic density plots for random forest imputation. (A) Ann Arbor stage. (B) B symptoms. Red line represents the distribution of observed values (before imputation); blue line represents distribution of imputed datasets. Close overlap between observed and imputed distributions confirms that imputation preserved the original data structure.

**Table S1:** Summary of demographic and clinical characteristics of 1548 patients with ALK‐positive ALCL.

**Table S2:** Sensitivity analysis of survival outcomes at different follow‐up time cutoffs.

**Table S3:** Univariate and multivariate Cox regression results for the overall patient cohort.

**Table S4:** Sensitivity analysis assessing robustness of imputation results.

## Data Availability

Data analyzed in this study were obtained from the publicly accessible, de‐identified SEER database (https://seer.cancer.gov/). The processed dataset and code used for data processing, statistical analysis, and model construction are available from the corresponding author upon reasonable request.
