# Supplementary figures and images for: Survival Trends and Prognostic Modeling in ALK‐Positive Anaplastic Large Cell Lymphoma: A Population‐Based Study in the Brentuximab Vedotin Era
Source: Cancer Med. 2026 Mar 6;15(3):e71695. doi: 10.1002/cam4.71695 (PMC12965843; doi:10.1002/cam4.71695)

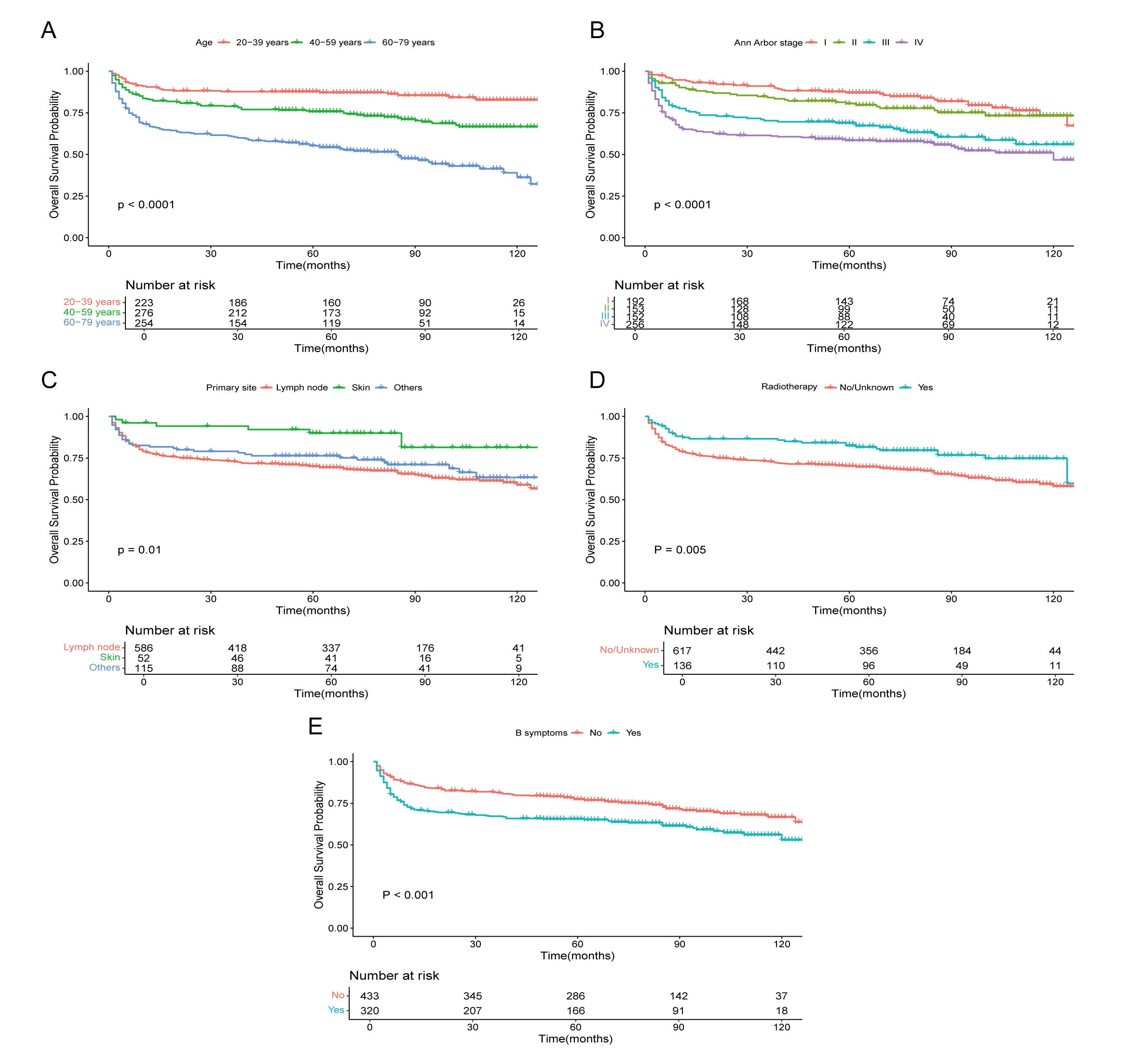

Supplement: Supplementary file 1 — Figure S1: Kaplan–Meier curves for key prognostic factors identified by the model (A) Age group: 20–39, 40–59, and 60–79 years. (B) Ann Arbor stage: I, II, III, and IV. (C) Primary site: lymph node, skin, and others. (D) Radiotherapy: no/unknown vs. yes. (E) B symptoms: no vs. yes. [file CAM4-15-e71695-s006.tif]

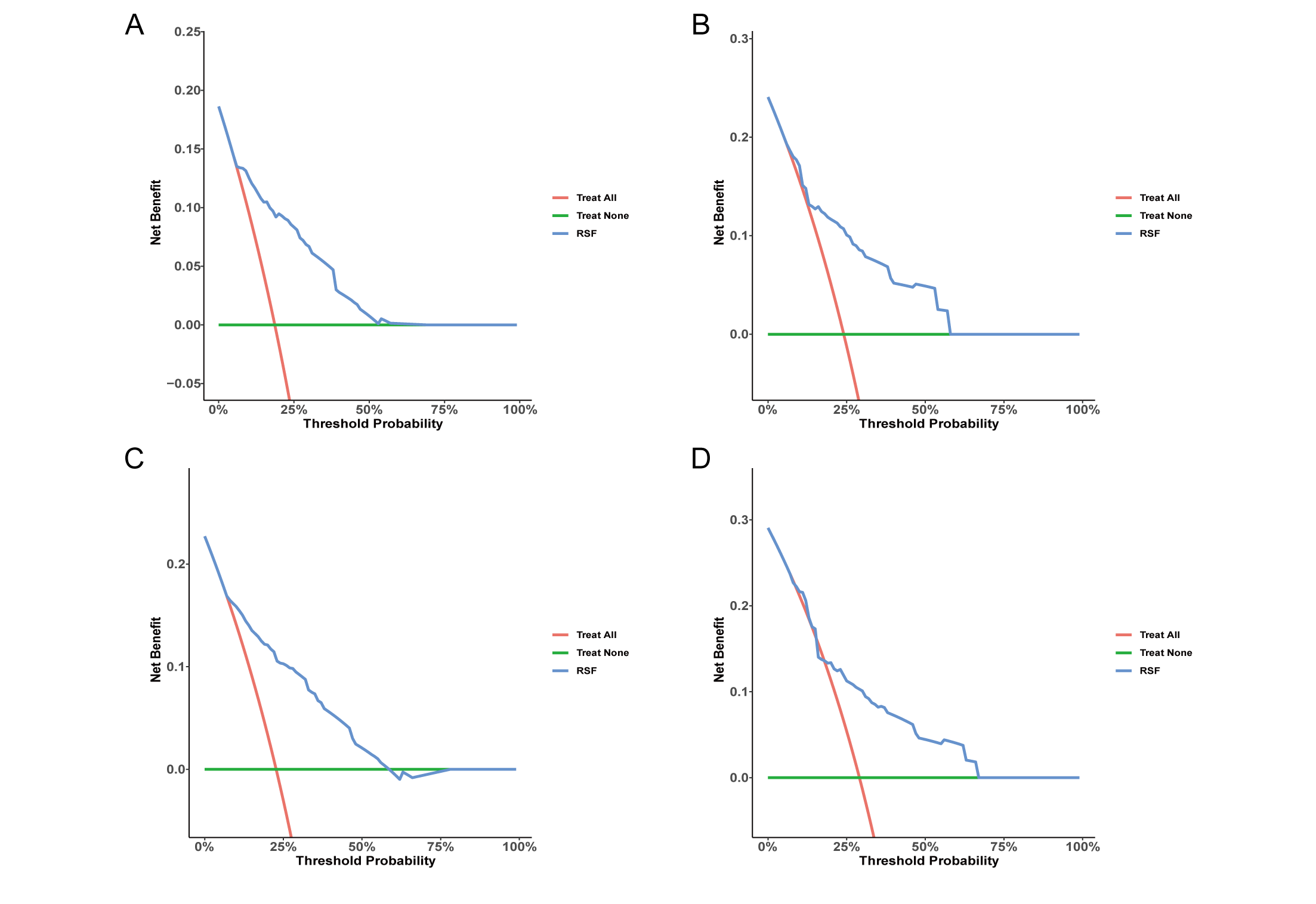

Supplement: Supplementary file 2 — Figure S2: Additional decision curve analysis (DCA) of the model (A–B) DCA curves for 1‐year OS in the training and testing cohorts (C–D). DCA curves for 3‐year OS in the training and testing cohorts. [file CAM4-15-e71695-s003.tif]

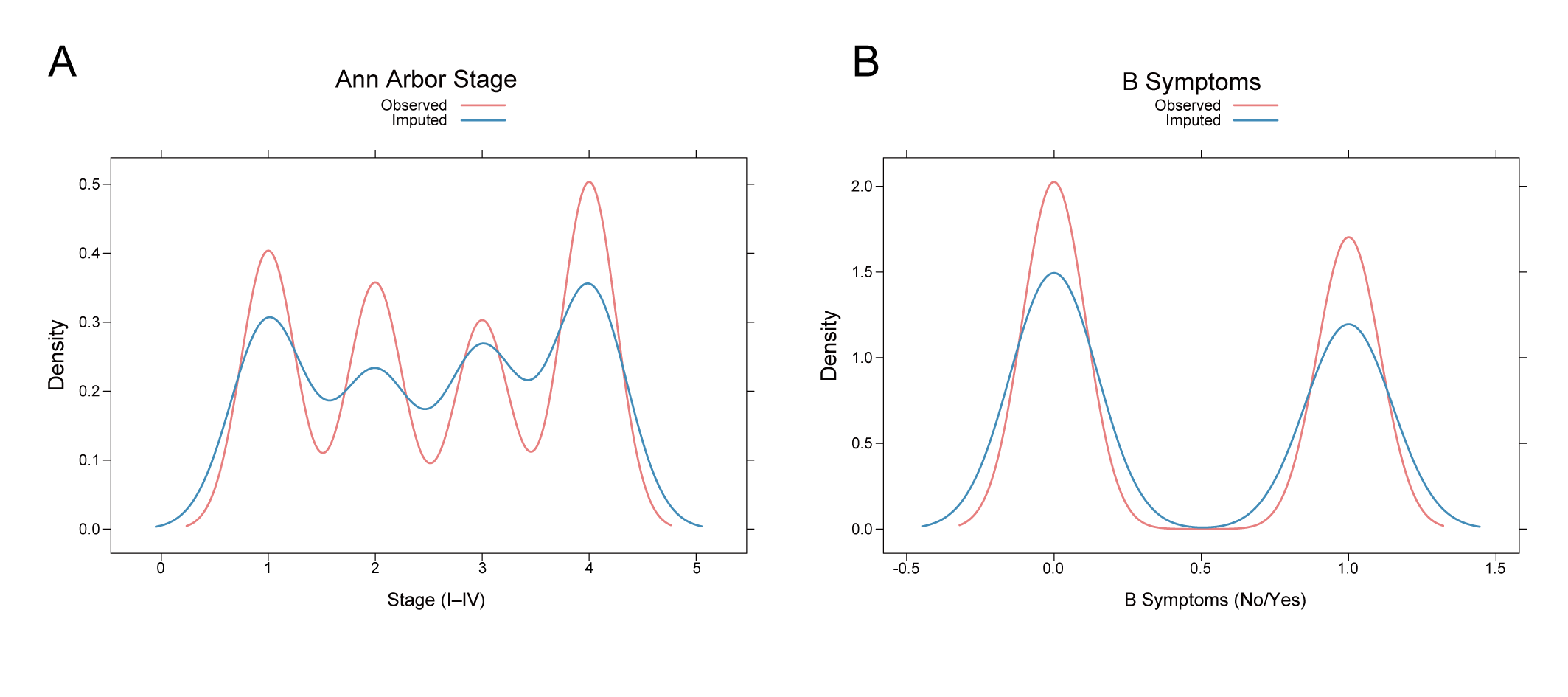

Supplement: Supplementary file 3 — Figure S3: Diagnostic density plots for random forest imputation. (A) Ann Arbor stage. (B) B symptoms. Red line represents the distribution of observed values (before imputation); blue line represents distribution of imputed datasets. Close overlap between observed and imputed distributions confirms that imputation preserved the original data structure. [file CAM4-15-e71695-s007.tif]
