# Supplementary material for: Survival Trends and Prognostic Modeling in ALK‐Positive Anaplastic Large Cell Lymphoma: A Population‐Based Study in the Brentuximab Vedotin Era
Source: Cancer Med. 2026 Mar 6;15(3):e71695. doi: 10.1002/cam4.71695 (PMC12965843; doi:10.1002/cam4.71695)
Supplement: Supplementary file 4 — Table S1: Summary of demographic and clinical characteristics of 1548 patients with ALK‐positive ALCL. [file CAM4-15-e71695-s002.docx]

Table S1. Summary of demographic and clinical characteristics of 1,548 patients with ALK-positive ALCL.

| Characteristic | Cases | % |
| --- | --- | --- |
| Age |  |  |
| 20–39 years | 437 | 28.2 |
| 40–59 years | 601 | 38.8 |
| 60–79 years | 510 | 32.9 |
| Sex |  |  |
| Female | 588 | 38.0 |
| Male | 960 | 62.0 |
| Year of diagnosis |  |  |
| 2004–2010 | 795 | 51.4 |
| 2011–2017 | 753 | 48.6 |
| Race |  |  |
| White | 1214 | 78.4 |
| Black | 205 | 13.2 |
| Others | 109 | 7.0 |
| Unknown | 20 | 1.3 |
| Primary site |  |  |
| Lymph node | 1171 | 75.6 |
| Skin | 125 | 8.1 |
| Others | 246 | 15.9 |
| Unknown | 6 | 0.4 |
| Ann Arbor stage |  |  |
| I | 332 | 21.4 |
| II | 294 | 19.0 |
| III | 249 | 16.1 |
| IV | 414 | 26.7 |
| Unknown | 259 | 16.7 |
| Radiotherapy |  |  |
| No/Unknown | 1230 | 79.5 |
| Yes | 318 | 20.5 |
| Chemotherapy |  |  |
| No/Unknown | 322 | 20.8 |
| Yes | 1226 | 79.2 |
| B symptoms |  |  |
| No | 704 | 45.5 |
| Yes | 592 | 38.2 |
| Unknown | 252 | 16.3 |

Abbreviations: ALCL, anaplastic large cell lymphoma.
