# Supplementary material for: Survival Trends and Prognostic Modeling in ALK‐Positive Anaplastic Large Cell Lymphoma: A Population‐Based Study in the Brentuximab Vedotin Era
Source: Cancer Med. 2026 Mar 6;15(3):e71695. doi: 10.1002/cam4.71695 (PMC12965843; doi:10.1002/cam4.71695)
Supplement: Supplementary file 5 — Table S2: Sensitivity analysis of survival outcomes at different follow‐up time cutoffs. [file CAM4-15-e71695-s001.docx]

Table S2. Sensitivity analysis of survival outcomes at different follow-up time cutoffs.

| Cutoff (Months) | Log-rank P | HR | 95%CI |
| --- | --- | --- | --- |
| 60 | < 0.001 | 0.63 | 0.53–0.76 |
| 84 | < 0.001 | 0.64 | 0.54–0.76 |
| 86 | < 0.001 | 0.67 | 0.56–0.79 |

Note: Follow-up was truncated at 60 months (5 years, standard oncology benchmark), 84 months (7 years), and 86 months (median follow-up of the post-BV cohort) to address differential follow-up duration between pre- and post-BV eras.

BV, brentuximab vedotin.
