# Supplementary material for: Survival Trends and Prognostic Modeling in ALK‐Positive Anaplastic Large Cell Lymphoma: A Population‐Based Study in the Brentuximab Vedotin Era
Source: Cancer Med. 2026 Mar 6;15(3):e71695. doi: 10.1002/cam4.71695 (PMC12965843; doi:10.1002/cam4.71695)
Supplement: Supplementary file 6 — Table S3: Univariate and multivariate Cox regression results for the overall patient cohort. [file CAM4-15-e71695-s004.docx]

Table S3. Univariate and multivariate Cox regression results for the overall patient cohort.

| Characteristic | Univariate analysis | | Multivariate analysis | |
| --- | --- | --- | --- | --- |
|  | HR (95% CI) | P | HR (95% CI) | P |
| Age |  |  |  |  |
| 20–39 years |  |  |  |  |
| 40–59 years | 2.01 (1.58-2.56) | < 0.001 | 2.09 (1.64-2.66) | < 0.001 |
| 60–79 years | 4.23 (3.35-5.34) | < 0.001 | 4.63 (3.66-5.86) | < 0.001 |
| Sex |  |  |  |  |
| Female |  |  |  |  |
| Male | 1.34 (1.14-1.58) | < 0.001 | 1.24 (1.05-1.46) | 0.011 |
| Year of diagnosis |  |  |  |  |
| 2004–2010 |  |  |  |  |
| 2011–2017 | 0.70 (0.60-0.83) | < 0.001 | 0.68 (0.58-0.81) | < 0.001 |
| Race |  |  |  |  |
| White |  |  |  |  |
| Black | 1.34 (1.08-1.66) | 0.007 | 1.34 (1.08-1.66) | 0.008 |
| Others | 1.15 (0.86-1.55) | 0.342 | 1.09 (0.81-1.46) | 0.587 |
| Primary site |  |  |  |  |
| Lymph node |  |  |  |  |
| Skin | 0.47 (0.33-0.67) | < 0.001 | 0.69 (0.47-1.03) | 0.068 |
| Others | 1.22 (1.00-1.49) | 0.048 | 1.38 (1.13-1.70) | 0.002 |
| Ann Arbor stage |  |  |  |  |
| I |  |  |  |  |
| II | 1.32 (1.03-1.70) | 0.030 | 1.34 (1.03-1.74) | 0.028 |
| III | 1.96 (1.54-2.50) | < 0.001 | 1.67 (1.28-2.18) | < 0.001 |
| IV | 2.55 (2.05-3.17) | < 0.001 | 2.18 (1.73-2.76) | < 0.001 |
| Radiotherapy |  |  |  |  |
| No/Unknown |  |  |  |  |
| Yes | 0.76 (0.62-0.92) | 0.006 | 0.88 (0.71-1.08) | 0.225 |
| Chemotherapy |  |  |  |  |
| No/Unknown |  |  |  |  |
| Yes | 1.02 (0.84-1.23) | 0.832 |  |  |
| B symptoms |  |  |  |  |
| No |  |  |  |  |
| Yes | 1.68 (1.44-1.95) | < 0.001 | 1.51 (1.28-1.78) | < 0.001 |
