# Supplementary material for: Survival Trends and Prognostic Modeling in ALK‐Positive Anaplastic Large Cell Lymphoma: A Population‐Based Study in the Brentuximab Vedotin Era
Source: Cancer Med. 2026 Mar 6;15(3):e71695. doi: 10.1002/cam4.71695 (PMC12965843; doi:10.1002/cam4.71695)
Supplement: Supplementary file 7 — Table S4: Sensitivity analysis assessing robustness of imputation results. [file CAM4-15-e71695-s005.docx]

Table S4. Sensitivity analysis assessing robustness of imputation results.

| Analysis | HR | 95% CI | P |
| --- | --- | --- | --- |
| Primary analysis | 0.68 | 0.58–0.81 | < 0.001 |
| Complete-case analysis | 0.66 | 0.54–0.80 | < 0.001 |

Note:

Primary analysis: full cohort after random forest imputation. Complete-case analysis: patients with no missing data in Ann Arbor stage or B symptoms. All models adjusted for the same covariates. Confidence intervals fully overlap, confirming robustness of findings.
